# Mechanical and thermal characterizations of nanoporous two-dimensional boron nitride membranes

**DOI:** 10.1038/s41598-022-10424-4

**Published:** 2022-04-15

**Authors:** Van-Trung Pham, Te-Hua Fang

**Affiliations:** 1 Department of Mechanical Engineering, National Kaohsiung University of Science and Technology, Kaohsiung, 807 Taiwan; 2 Department of Mechanical Engineering, Pham Van Dong University, Quang Ngai, 570000 Vietnam

**Keywords:** Mechanical properties, Nanoscale materials, Two-dimensional materials

## Abstract

Hexagonal boron nitride (h-BN) is a promising 2D material due to its outstanding mechanical and thermal properties. In the present study, we use molecular dynamics simulations to investigate the influence of porosity and temperature on the mechanical characteristics of h-BN based on uniaxial and biaxial tensions. Meanwhile, the progression of the microstructure of h-BN up to fracture is studied in order to clarify its fractures mechanism during the tension process. Our results reveal that depending on the porosity and tensile direction, the phase transition occurs more or less. The strength, and Young's modulus of h-BN membranes reduce as increasing porosity. Due to the presence of the pores, the most substantial stresses will be centred around the pores site in the tensile test. Then the fracture starts on the pore edge and spreads preferentially along the zigzag direction of h-BN. Furthermore, fracture strain, strength, and Young's modulus decrease when the temperature rises. In addition, the non-equilibrium molecular dynamics (NEMD) simulations are performed to investigate the influence of various porosities and temperatures on the thermal conductivity of h-BN membranes. The results reveal that the thermal conductivity is greatly reduced by nanoporous. The higher the porosity, the lower the thermal conductivity. The vibration density of states of h-BN membranes is calculated; the result suggests that the defects might reduce the phonon mean free path because of the high collision of the phonons. These alterations represent the scattering influence of defects on phonons, which reduces phonon life and considerably lowers thermal conductivity. Moreover, the findings also proved that as temperature increases, the intrinsic thermal conductivity of h-BN decreases. The thermal conductivity and mechanical properties of the pristine h-BN thin film are interestingly equivalent in the zigzag and armchair orientations.

## Introduction

Since the discovery of graphene in 2004^1^, its intriguing features have piqued the interest of researchers all over the globe in other two-dimensional (2D) nanomaterials for a variety of applications. Porous 2D materials with changeable pore shape and size have attracted a lot of attention because of their potential uses in a variety of applications, including photocatalysis, electrocatalysis, biomaterials science, energy generation, storage^2–4^. Porous materials may interact with a variety of active species both within their porous frameworks and on their surface, but only if these substances fit the pore size of porous materials, which is crucial for gas storage, separation, adsorption, and heterogeneous catalysis^5,6^. As a result, several methodologies for fabricating porous nanostructures containing functional building blocks have been investigated earlier^7,8^.

Nowadays, with the development of nanotechnology and advanced manufacturing, it is possible to fabricate nanomaterials with the porosity as well as defects to be adjusted according to the requirements^9–11^. For 2D nanomaterials, the techniques of bottom-up^12^, laser scribing^13^, and plasma irradiation^14^ allow for the creation and engineering of pores with dimensions smaller than a nanometer. Moreover, porous engineering might be a useful technique for tuning material behavior. By carefully controlling point defects, many advanced materials acquire their superior properties. The thermal conductivity of materials may be systematically controlled and optimized by carefully manipulating nanoporous^15^. Because of their distinctive structure and characteristics as well as intriguing applications, porous 2D materials including graphene, borophene, metal chalcogenides, metal oxides, and carbon nitride have gotten a lot of interest in recent years^16–19^.

Hexagonal boron nitride (h-BN) is a promising 2D material with excellent mechanical, chemical, and thermal characteristics^20^. Single-layer hexagonal boron nitride is structurally similar to graphene but with alternating boron and nitrogen atoms instead of carbon. Graphene and h-BN have been presented as options for the fabrication of heterostructures with customizable physical properties due to their atomic structure similarities^21^. Because of its geometric resemblance to graphene, h-BN possesses several desirable physical qualities, such as excellent mechanical properties and good chemical and thermal stability^22^. Furthermore, h-BN has a number of unique characteristics that are not seen in graphene. For instance, without any external electronic field modulation, h-BN shows intrinsic half-metallicity^23^. Falin et al.^24^ have investigated the indentation process of h-BN nanosheets, and they reported that the mechanical properties of few-layer BN differ significantly from those of few-layer graphene. Unlike graphene, which loses more than 30% of its strength as the number of layers grows from 1 to 8, the mechanical strength of BN nanosheets is unaffected by increasing thickness. This nanomaterial also has an exceptional ultraviolet optical property^25,26^. The h-BN has many other excellent properties which are beneficial for their applications, such as lubrication^27–29^, low dielectric parameter for microwave absorption^30–32^, high thermal conductivity^33–35^, adsorption^36–38^, and so on. These remarkable features have prompted h-BN research to become one of the fascinating topics in nanoscience today. Recently, some researchers have studied the mechanical and heat transport properties of h-BN. For instance, using experimental supported by theoretical analysis, Yang et al.^39^ reported that the crack growth in h-BN is surprisingly stable. The findings point to further practical benefits and new technical potential for monolayer h-BN, such as mechanical protection for 2D devices. Using density functional theory calculations, Wu et al.^40^ report on the mechanical characteristics of h-BN and its band structures adjusted by straining. According to their findings, the strain may adjust single-layer h-BN from an insulator to a semiconductor. Mortazavi et al.^41^ conducted extensive molecular dynamics (MD) simulations to study the effects of grain size on the thermal characterization of polycrystal h-BN monolayer. The findings showed that the grain boundary resistance accounts for the majority of the sample's thermal resistance. However, increasing the grain size significantly reduces the boundary thermal resistance effect. In addition, there are many studies on the mechanical and thermal characteristics of the heterostructure of h-BN and graphene^42,43^. Li et al.^42^ used the molecular dynamics method in combination with the density functional theory and classical disclination theory to study the mechanical properties of grain boundaries (GBs) in planar heterostructures of graphene and hexagonal boron nitride.

As previously stated, a deeper knowledge of the structure–property relationship is critical for structural design and function optimization in a wide range of technical applications. However, the effect of nanopores uniformly dispersed on single-layer h-BN has received little attention. The influences of porosity and temperature on the biaxial tensile test, in particular, have yet to be fully investigated. As a result, research into their impact on the mechanical and thermal characterizations of nanoporous 2D h-BN membranes is required.

Motivated by the above discussion, this study focuses on the effects of porosity and temperature on thermal conductivity and the mechanical characteristics of nanoporous h-BN membranes.

## Method

### Tensile properties calculations

**Figure 1:**
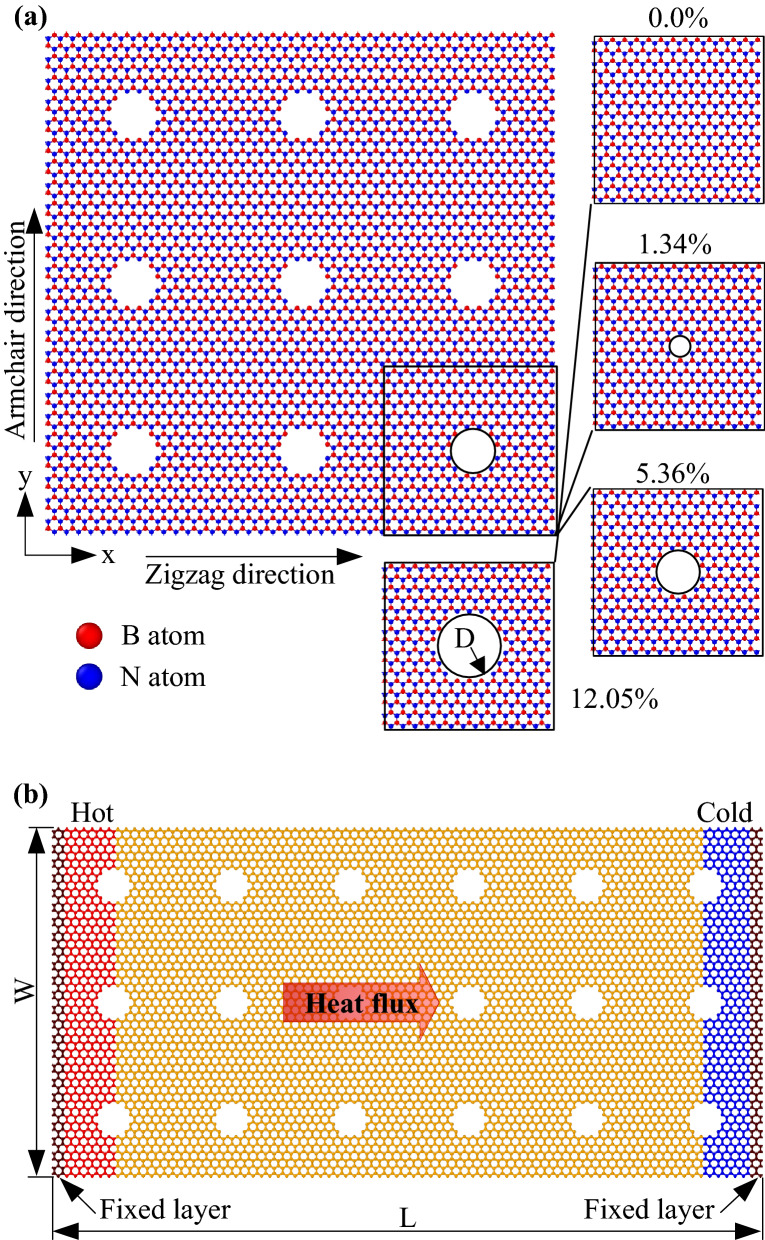
**(a)** Atomistic configuration of monolayer h-BN, and **(b)** Schematics of the non-equilibrium MD simulation of monolayer h-BN.

To investigate the effect of porosity and temperature on mechanical properties of monolayer h-BN, a model of monolayer h-BN with various porosities is created, as illustrated in Fig. 1a. The size of the models is approximately 10.5 × 10.5 nm^2^ along *x-* and *y-*directions. The vacuum region is set as 5.0 nm above and below the monolayers along *z-*direction to minimize the interaction between the membranes. In order to eliminate the influence of the simulation box boundaries, periodic boundary conditions are applied in all dimensions. The porosity is changed with the values of 0.0% (non-defective), 1.34%, 5.36%, 12.05%, where porosity is defined by the ratio of missing atoms to total atoms of the pristine sheet. The pores in the membranes are circular pores with the diameter of circular pores ranging from 4.35 Å to 13.05 Å. To investigate the tensile mechanical properties of h-BN, we used the LAMMPS software to run molecular dynamics simulations^44^. The Tersoff potential created by Kınacı et al.^45^ was used to represent the atomistic interactions between B-B, N–N, and B-N atoms in the MD simulations. This potential has been verified to be suitable for studying the mechanical properties and heat transfer of h-BN with previous studies^46^. All initial configurations were relaxed by the conjugate gradient (CG) method to achieve an equilibrium minimum energy. Then, all systems were relaxed by equilibrating in an isothermal-isobaric (NPT) ensemble for 100 ps at target temperatures (100, 200, 300, 400, 500, and 600 K) and zero pressure. The time-step is set to 0.5 fs. After equilibration, we applied the uniaxial or biaxial tension with a constant strain rate of 5.10^8^ s^-1^ along with the deformation directions (armchair and zigzag). Here, the engineering strain was defined as *ε* = *(L-L*_*0*_*)/L*_*0*_, where *L*_*0*_ and *L* are the lengths of monolayer h-BN before and after the deformation. The atomic stress in the simulation system is calculated using the virial stress formulation:

1 $$ \sigma = \frac{1}{V}\sum\limits_{i \in V} {\left[ { - m_{i} v_{i} \otimes v_{i} + \frac{1}{2}\sum\limits_{j \ne i} {(r_{ij} \otimes F_{ij} )} } \right]} $$

where, *V* is the volume of the monolayer h-BN with a thickness of 0.33 nm. *m*_*i*_ and *v*_*i*_ denote the mass and the velocity vector of the atom *i-*th. *r*_*ij*_ and *F*_*ij*_ represent the distance vector and the force between particle *i* and particle *j*. The symbol $\otimes$ denotes the tensor product of two vectors.

The von Mises stress *σ*_*von*_ is given by:

2 $$ \sigma_{von}^{2} = \frac{1}{2}\left[ {\left( {\sigma_{xx} - \sigma_{yy} } \right)^{2} + \left( {\sigma_{yy} - \sigma_{zz} } \right)^{2} + \left( {\sigma_{zz} - \sigma_{xx} } \right)^{2} + 6\left( {\sigma_{xy}^{2} + \sigma_{yz}^{2} + \sigma_{zx}^{2} } \right)} \right] $$

The OVITO visualization software is used to observe the evolution of the atomic structure and process data achieved from the MD simulations^47^.

### Thermal conductivity calculations

We used the schematics to compute the thermal conductivity of the h-BN membranes, as shown in Fig. 1b. The simulated structures are *W* × *L*, where *L* is the dimension of the system along the heat flux direction (armchair or zigzag directions), and *W* is the width of the simulated structure. We used the NEMD method to calculate the thermal conductivity of h-BN membranes. The length of samples has a considerable impact on the thermal conductivity measurements obtained using the NEMD approach^48^. Then, we change the length in the range of 21.1, 31.6, 42.2, 63.3, and 84.4 nm in the heat transfer direction, while the width is kept the same at *W* = 10.5 nm. The structure has the same porosity as the model in Fig. 1a. The periodic boundary condition is employed in the width direction to avoid the effect of the width dimension, while the fixed boundary condition is employed in the heat transport direction. The model was divided into 50 slabs, and the heat sink and source were adjacent to the fixed atoms at both ends. All simulations were run with a time step of 0.5 fs. All systems were relaxed by equilibrating in an NVT ensemble for 500 ps. After the full relaxation, the system is switched to NVE (constant number of atoms, volume, and energy) ensemble for 500 ps, and we apply the Langevin thermostats to the heat source and sink to obtain steady-state heat flux *J* by exchanging the kinetic energies between the heat source and sink for 5.0 ns*.* The heat source and sink were controlled at *T*_*H*_ = *T(1* + *Δ)* K and *T*_*C*_ = *T(1-Δ)* K, here *Δ* = 0.05, and *T* is the predefined temperature. *T* ranged from 100 to 600 K. Once the system becomes steady-state, a stable temperature gradient along the heat transport direction is achieved, the heat flux *J* can be calculated:

3 $$ J = \frac{dE/dt}{A} $$

where A is the cross-section area that the heat flux passes through with the thickness of monolayer h-BN is chosen as 0.33 nm^49^. *dE/dt* stands for the rate of adding or removing kinetic energy in the thermostat regions.

The thermal conductivity *κ* is calculated via the Fourier’s law:

4 $$ \kappa = \frac{J}{dT/dx} $$

where (*dT/dx*) is the time-averaged temperature gradient along the heat transfer direction.

## Results and discussion

### Tension

**Figure 2:**
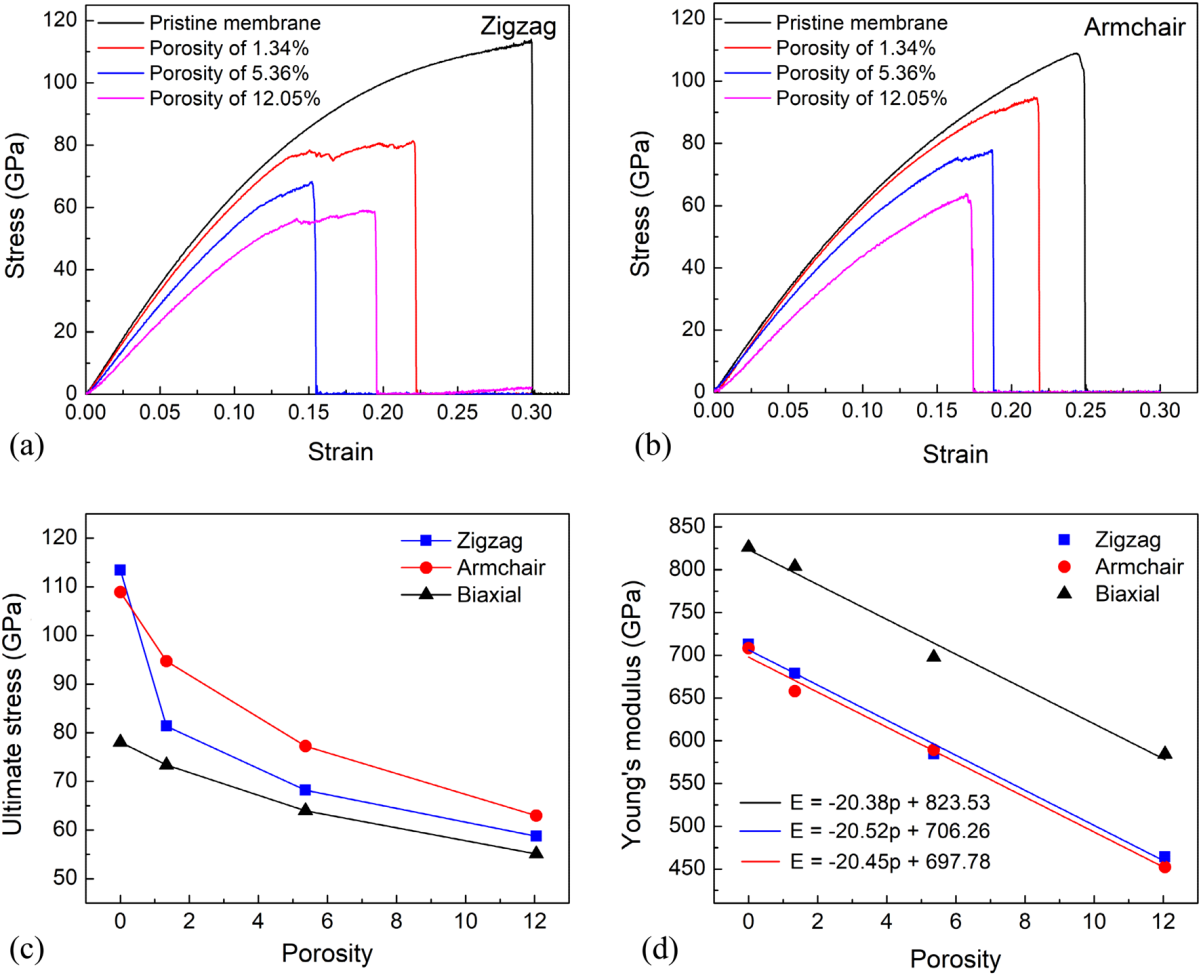
Tensile characteristics of the h-BN membrane with various porosities at a temperature of 300 K. (**a**) Stress–strain curves of the h-BN membrane for strain applied along the zigzag direction. (**b**) Stress–strain curves of the h-BN membrane for strain applied along the armchair direction. (**c**) The porosity dependence of ultimate stress. **(d)** The porosity dependence of Young's modulus.

We begin our discussion by studying the tensile properties of the h-BN membranes with different porosities under tension, as shown in Fig. 2. The stress–strain curves of the h-BN nanosheet for strain applied along the zigzag and armchair directions are illustrated in Fig. 2a,b, respectively. The stress–strain curves of the h-BN membrane with various porosity at a temperature of 300 K in biaxial tension are illustrated in Fig. S1. In every example, three distinct behaviors in the stress–strain response were found. As can be seen, a linear relationship occurs at low strain levels, and after that, a non-linear response up to the ultimate strength, followed by a sudden fall in stress that corresponds to membrane rupture. In the non-linear response region, phase transition may exist. Depending on the porosity and tensile direction, this phase transition occurs more or less. For example, for a membrane with a porosity of 1.34% under uniaxial in the zigzag direction, the phase transition occurs when the strain ranges from 13.93% to 22.09%, as shown in Fig. 2a, and more details on the phase transition of this membrane are depicted in Figs. 4 and 5a. For the membrane with a porosity of 12.05% under uniaxial in the zigzag direction, the phase transition occurs as the deformation is from 14.05% to 18.05% as shown in Fig. 2a, and more details on the phase transition of this membrane are depicted in Fig. 5c. While tensing in the armchair direction, the phase transition is very rare, as depicted in Figs. 2b and 6a1–c1. Fig. S1 and Fig. S2 show that the phase transition before the nanosheet is destroyed is rare under biaxial tension. The porosity dependence of ultimate strength is determined from the stress–strain curves and plotted in Fig. 2c. It shows that the strength reduces with the increased porosity in both uniaxial and biaxial tensions. However, like prior investigations for graphene sheets and borophene^19,50,51^, the fracture strain value appears to be insensitive by the material's porosity. The strength of the pristine h-BN monolayer under uniaxial tension in the zigzag orientation is quite close to that in the armchair orientation. However, with porous sheets, the strength of h-BN sheets when stretched in the armchair direction is higher than when stretched in the zigzag direction. This evidences that under uniaxial tension in the zigzag direction, the strength of the h-BN sheet is more sensitive to voids than stretched in the armchair direction. Furthermore, the strength of the h-BN nanosheet under biaxial tension is the smallest in all cases. Young's modulus dependence of porosity is calculated from stress–strain curves and plotted in Fig. 2d. Similar to strength, Young's modulus also reduces as increasing porosity. Biaxial tension has a greater modulus than zigzag or armchair tension, which agrees with the phenomenon of graphene material^52^.

From the porosity dependence of Young's modulus values of the h-BN membranes, we can build a linear equation of Young's modulus *E* according to the porosity *p* with a coefficient of determination *R*^2^ as follows:

5 $$ {\text{For}}\;{\text{biaxial}}\;{\text{tension: }}E = - 20.38p + 823.53\;({\text{GPa}})\quad {\text{with}}\;{\text{R}}^{{2}} = 0.{9844} $$

6 $$ {\text{For}}\;{\text{zigzag}}\;{\text{direction}}:E = - 20.52p + 706.26\;({\text{GPa}})\quad {\text{with}}\;{\text{R}}^{{2}} = 0.{9915} $$

7 $$ {\text{For}}\;{\text{armchair}}\;{\text{direction: }}E = - 20.45p + 697.78\;({\text{GPa}})\quad {\text{with}}\;{\text{R}}^{{2}} = 0.{9893} $$

In general, the results show that the lower the porosity, the higher the mechanical properties.

**Figure 3:**
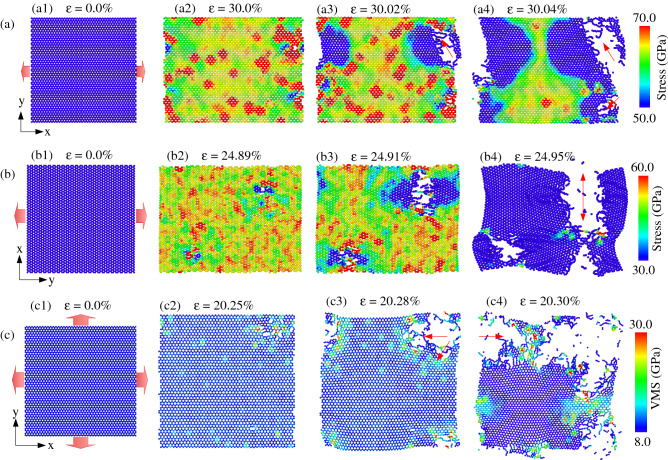
**(a)** The stress distribution and fracture behavior of single-layer h-BN under uniaxial tension along the *x* (zigzag) direction at a temperature of 300 K. **(b)** The stress distribution and fracture behavior of single-layer h-BN under uniaxial tension along the *y* (armchair) direction at a temperature of 300 K. **(c)** The fracture evolution and the von Mises stress distribution of h-BN membrane under biaxial tension at 300 K.

Figure 3a shows the stress distribution and deformation behavior of monolayer h-BN under uniaxial tension in the zigzag direction at 300 K. The atoms of the nanosheet are colored according to the normal stress (*σ*_*xx*_). The red color represents high stress, and the blue color denotes low stress. We can see that as the strain raises, the stress in the membrane raises. As the strain value gets to 30%, the crack begins to appear on the sheet. This crack rapidly expands until the nanosheet is entirely shattered at a strain value of 30.04%. Intriguingly, the fracture is not perpendicular to the tensile orientation but tends to propagate in the red arrow direction, the direction of the zigzag edge. The stress distribution and deformation behavior of monolayer h-BN during uniaxial tension along the *y* (armchair) direction at 300 K are presented in Fig. 3b. The membrane atoms are colored according to normal stress (*σ*_*yy*_). We can see that the crack begins to appear on the nanosheet when the strain value reaches 24.89%. This crack rapidly expands until the sheet is completely shattered at a strain value of 24.95%. Unlike when tensing in the zigzag direction, when tensing in the armchair direction, the fractures spread perpendicular to the tensile direction. It is interesting to see that the fractures preferentially spread in the zigzag edge under deformation in the zigzag or armchair direction. The priority of crack spreading along the zigzag edge of the h-BN nanosheet is similar to that of other 2D materials such as graphene, MoS_2_^53–55^. The von Mises stress distribution and the fracture progress of the h-BN membrane under biaxial tension at 300 K are illustrated in Fig. 3c. All atoms on the membrane are coloured according to their VMS values; the higher VMS is red, while the lower VMS is blue. As the strain increases, the VMS on the membrane rises. When the strain reaches 20.25 percent, the membrane begins to rupture. Cracks spread quickly along the red arrow directions as strain increases (zigzag direction). Following the red arrows, these breaks progressively spread throughout the membrane until it is completely destroyed. Cracks that grew in the zigzag orientation (red arrows) were more common than those that occurred in the armchair orientation. The fracture morphology of the membrane under biaxial tension is more rough and convoluted than it is under uniaxial tension. Under biaxial tension for the sheets with different porosity, crack propagation along the zigzag edge also dominates, as shown in Fig. S2.

**Figure 4:**
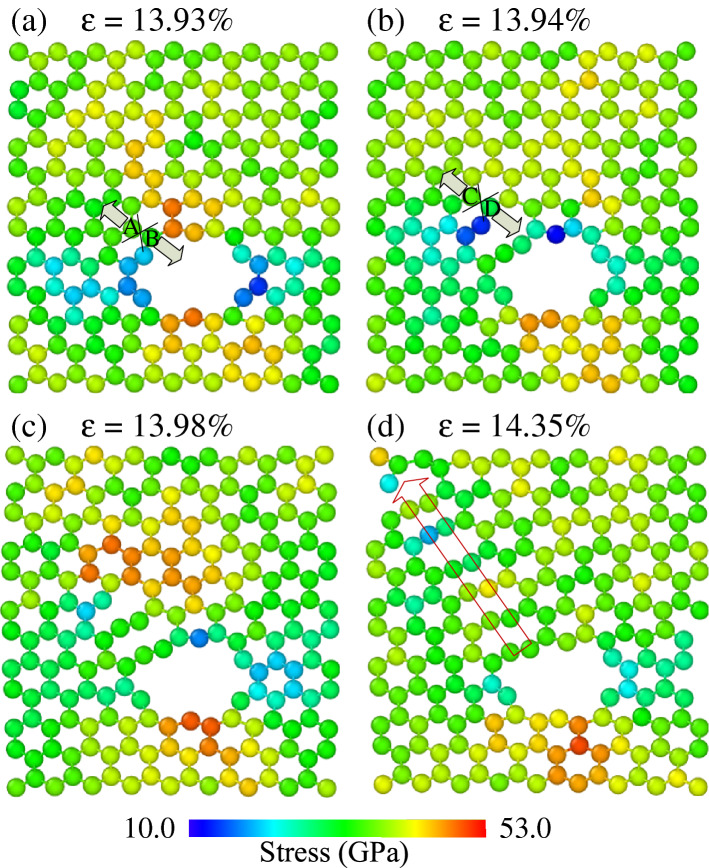
Representative MD snapshots of the h-BN for the porosity of 1.34% at different strain percentages: (**a**) 13.93%, (**b**) 13.94%, (**c**) 13.98%, (**d**) 14.35% (scale bar indicates the magnitude of the normal stress *σ*_*xx*_).

**Figure 5:**
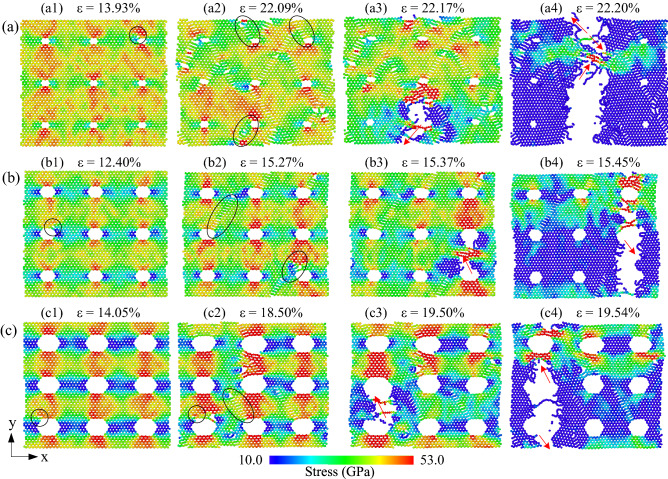
The stress distribution and fracture evolution of h-BN membranes in uniaxial tension along the zigzag direction at 300 K with different porosities: (**a**) 1.34%, (**b**) 5.36%, (**c**) 12.05%.

Figure 4 shows the phase transition of the h-BN membrane with a porosity of 1.34% during uniaxial tension in the zigzag direction. The atoms of the nanosheet are colored according to the normal stress *σ*_*xx*_. As shown in the diagram, Fig. 2a shows that with increasing strain, the stress increases gradually. When the strain reaches 13.93%, the tensile stress in the zigzag direction begins the inelastic regime. At the strain value of 13.93%, the bond between atoms A and B reaches the critical value and begins to break, thus causing the beginning of phase transition from six-membered ring to ten-membered ring, as depicted in Fig. 4a,b. As strain value of 13.98%, the bond between atom C and atom D is broken, as depicted in Fig. 4c. The phase transition from six-membered to ten-membered ring continuously happens as the strain percentage increases. Interestingly, this phase transition is propagated along the zigzag edge, as shown in Fig. 4d. This process continues until the plate reaches the critical stage and enters the failure phase, as shown in Fig. 5a2.

Figure 5 shows the deformation and fracture process of monolayer h-BN during uniaxial tensile along the zigzag direction at 300 K with different porosities. Figure 5a1–c1 show the stress distribution and h-BN membrane structure when the membranes begin to have phase transitions. We divide the sheet into many slabs along the tensile direction. At the slab containing the holes, the bonding density in the cross-section area is reduced, resulting in high stresses concentrated in this slab. It shows that the high stress is concentrated in the atoms right at the hole position and in the slab containing the holes. As increasing the strain, the sheet has undergone a phase transition similar to Fig. 4. The phase transition occurs along the zigzag edge (shown in black ellipses). Figure 5a2–c2 show the stress distribution and h-BN membrane structure at the strain value that the sheet begins to shatter. As the strain increases, the structure of the sheets is shattered in the red arrow direction, as shown in Fig. 5a3–c3. At the same time, the phase transition continues because high stresses are concentrated at the edge of the fracture. The phase transition and crack propagate along the red arrows until the membrane is completely ruptured, as shown in Fig. 5a4–c4.

**Figure 6:**
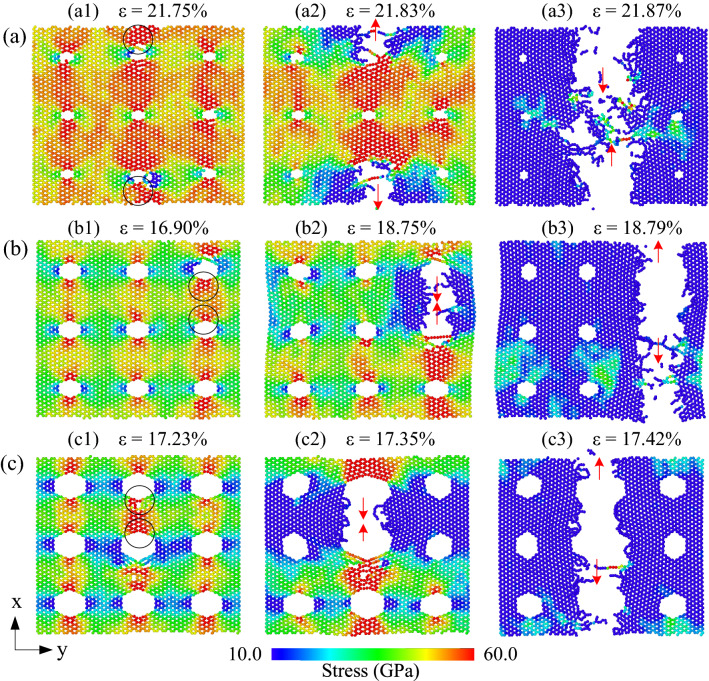
The stress distribution and fracture evolution of h-BN membranes in uniaxial tension along the armchair direction at 300 K with different porosities: (**a**) 1.34%, (**b**) 5.36%, (**c**) 12.05%.

The stress distribution on the h-BN membrane when stretched in the *y*-direction (armchair direction) for different porosities at 300 K is shown in Fig. 6. The atoms are coloured according to the atomic-level stress tensor σ_yy_ in armchair tension. In which blue represents the atoms with low stress, red indicates high-stress atoms. It shows that the atomic density is low at the cross-sections with holes, leading to high-stress concentration at this low atomic density cross-section. When the sheets reach the ultimate tensile stress, as shown in Fig. 6a1–c1, the atoms located at the edge of the nanopore have the highest stress value (shown in black circles). As strain increases, generally, the crack is initiated on the edge of the nanopore, which rapidly propagates along the red arrow. The appearance and propagation of the crack reduce local stress. Eventually, these cracks rapidly propagate through the entire neck, and the sheet is completely destroyed. The results show that when tensing in the armchair direction, the cracks propagate in the zigzag direction. That is, the crack propagates along the zigzag edge. The fractures preferentially propagate and migrate along zigzag edges of h-BN sheets. As illustrated in Figs. 5 and 6, the sharp corners of the nanopore cause stress concentration in the tensile test, and the corners are commonly the first site where the fracture occurs.

**Figure 7:**
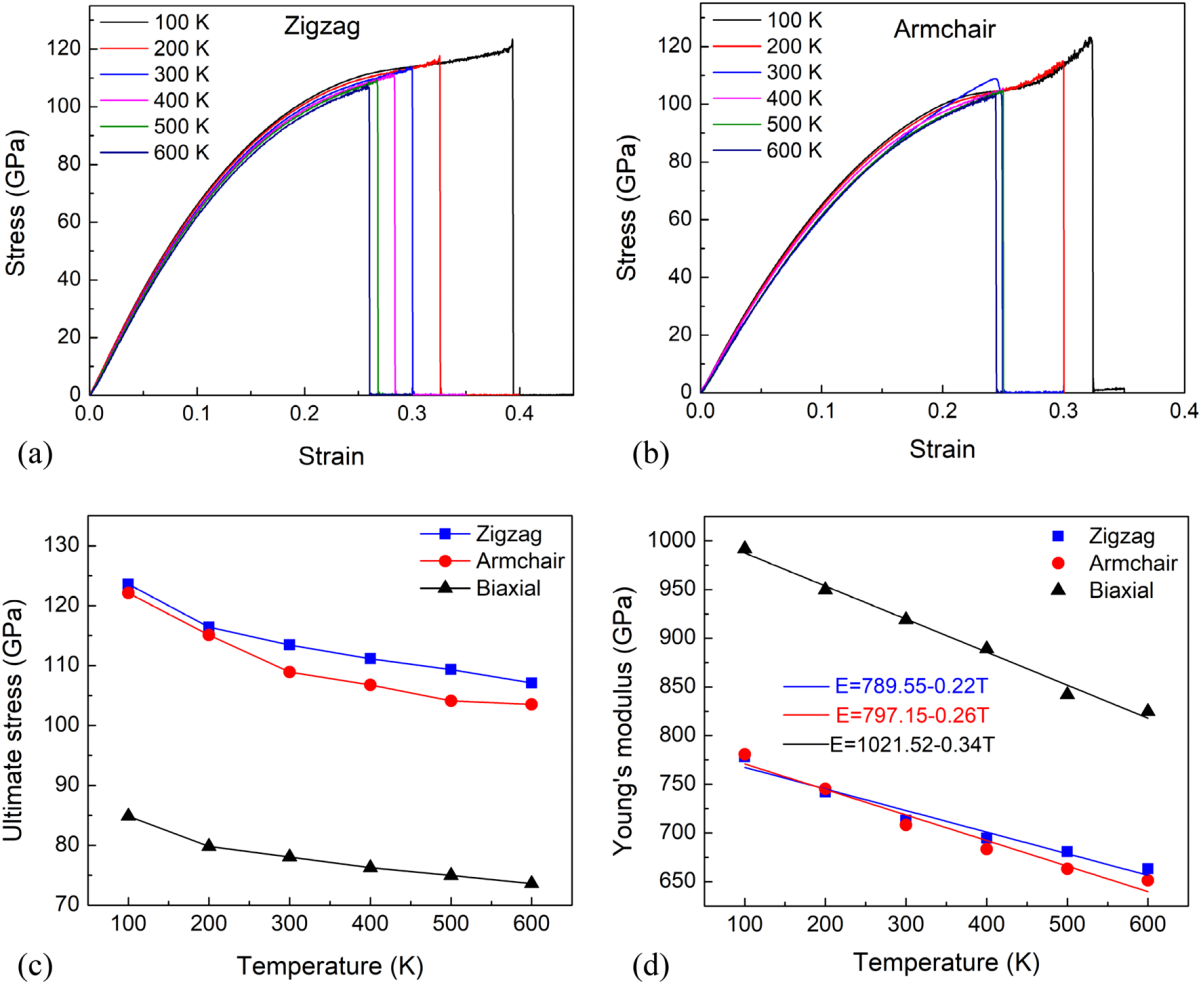
(**a**) The stress–strain relations of the pristine h-BN membrane under uniaxial tension along the zigzag direction with different temperatures. (**b**) The stress–strain relations of the pristine h-BN membrane under uniaxial tension along the armchair direction with different temperatures. (**c**) The temperature dependence of ultimate strength. **(d)** The temperature dependence of Young's modulus.

With different temperatures, the stress–strain relationship of the h-BN membrane under the uniaxial tensile test in zigzag and armchair directions are illustrated in Fig. 7a,b, respectively. The stress–strain curves of the h-BN membrane under the biaxial tensile test at various temperatures are exhibited in Fig. S3. In every example, three distinct behaviors in the stress–strain response were found. It shows that a linear relationship occurs at low strain levels; after that, a non-linear response up to the ultimate strength, followed by a sudden fall in stress that corresponds to specimen rupture. The fracture strain decreases with the increase of temperature in zigzag tension and biaxial tension. However, in the uniaxial tension in the armchair direction, the fracture strains are close to each other when the temperature is above 300 K. From the stress–strain relationships, the temperature dependence of ultimate strength and the temperature dependence of Young's modulus are determined and plotted in Fig. 7c and d, respectively. The result depicts that the ultimate strength and Young's modulus of h-BN membrane reduce with the increased temperature in both uniaxial and biaxial tensions. It emphasizes that temperature has a significant influence on the stress–strain relationship. As the temperature rises, the temperature-induced softening causes mechanical characteristics to decrease^56–59^. The strength of the pristine h-BN membrane under uniaxial tension along the zigzag direction is quite close to that in the armchair direction. Under the biaxial tension, the strength of the h-BN membrane is greatly reduced compared to uniaxial tension. Similar to strength, Young's modulus also decreases as the temperature rises. At different temperatures, we find that Young's modulus value of the h-BN membrane is larger in the biaxial tensile test than in the uniaxial tensile test, which is in good agreement with the phenomenon of graphene material^52^.

From the temperature dependence of Young's modulus of the h-BN membrane, we can build a linear equation of Young's modulus *E* according to the temperature *T* with a coefficient of determination *R*^2^as follows:

8 $$ {\text{For}}\;{\text{zigzag}}\;{\text{direction}}:E = - 0.22T + 789.55\;({\text{GPa}})\quad {\text{with}}\;{\text{R}}^{{2}} = 0.{9557} $$

9 $$ {\text{For}}\;{\text{armchair}}\;{\text{direction}}:E = - 0.26T + 797.15\;({\text{GPa}})\quad {\text{with}}\;{\text{R}}^{{2}} = \, 0.{9575} $$

10 $$ {\text{For}}\;{\text{biaxial}}\;{\text{tension}}:E = - 0.34T + 1021.52\;({\text{GPa}})\quad {\text{with}}\;{\text{R}}^{{2}} = 0.{9884} $$

The impact of strain rate on h-BN mechanical characteristics is also examined. We choose the strain rates in the range of 5 × 10^7^ to 5 × 10^9^ s^-1^, commonly employed in MD simulations, to investigate the strain rate impact. Fig. S4 illustrates the stress–strain curves of the pristine h-BN membrane under the uniaxial tension along the zigzag and armchair directions at a temperature of 300 K. Before the rupture happens, the stress–strain curves at all strain rates are almost identical, demonstrating that strain rate has only a minor influence on Young's modulus of h-BN. The result shows that increasing strain rate leads to the ultimate strength and failure strain increase. Because the atoms have less time to respond to the loading when the strain rate is higher; as a result, the broken bonds are homogeneously distributed, leading to a higher fracture strength and strain. Overall, the influence of strain rate on the fracture strain and ultimate strength of h-BN is relatively weak compared to the influence of porosity and temperature.

### Thermal conductivity

The effects of porosity and temperature on the thermal conductivity of nanoporous h-BN membranes are explored in this section. The intrinsic thermal conductivity (thermal conductivity at infinite length) is calculated in two steps: following a series of size-dependent simulations, a size-independent extrapolation is done. As a result, we will simulate different lengths for each porosity and temperature to compute the intrinsic thermal conductivity value.

**Figure 8:**
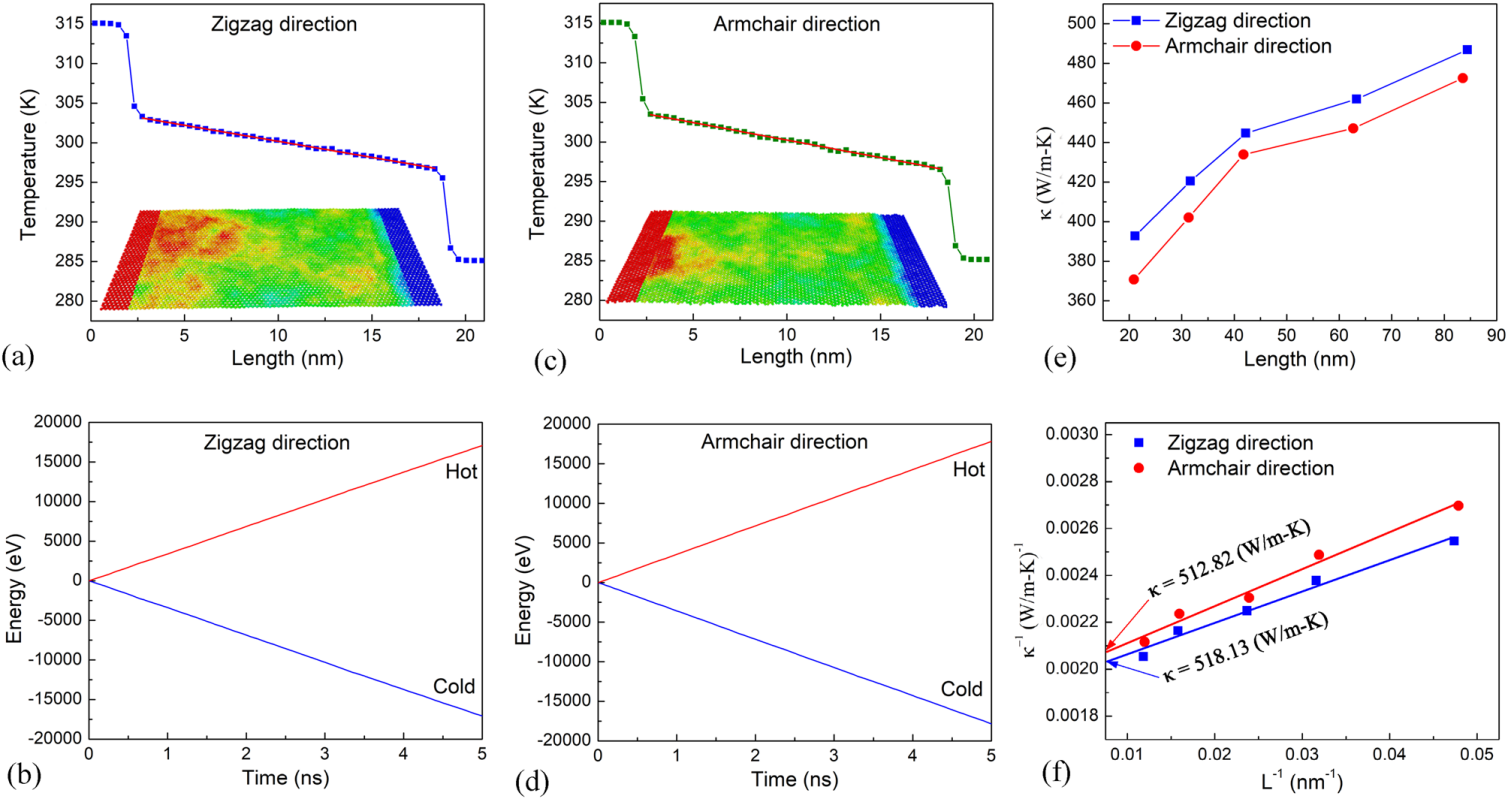
(**a**) The temperature profile for the monolayer h-BN membrane with L = 21.1 nm under the heat transfer along the zigzag direction. (**b**) The thermal energy that removed from the heat sink and added to the heat source in the heat transfer process along the zigzag direction. (**c**) The temperature profile for the monolayer h-BN membrane with L = 20.9 nm under the heat transfer along the armchair direction. (**d**) The thermal energy that removed from the heat sink and added to the heat source in the heat transfer process along the zigzag direction. **(e)** Dependence of thermal conductivity on the system size of monolayer h-BN in zigzag and armchair directions at temperature of 300 K. **(f)** Linear fitting of *1/κ* and *1/L* for zigzag and armchair directions.

Figure 8a,c presents the temperature profile of the h-BN membrane obtained using the NEMD at 300 K in the zigzag and armchair directions, respectively. The red linear in Fig. 8a,c represents the steady-state temperature profile. The computed energies added to the heat source and removed from the heat sink according to time are shown in Fig. 8b,d. As shown, the amount of energy removed from the cold reservoir equals the quantity of energy given to the hot reservoir, implying that the system's total energy is precisely preserved. These findings suggest that the system's energy remains constant and that steady heat flux is provided throughout the system. At each simulation time step, this heat flux is a constant amount of kinetic energy flowing from the atoms in the hot reservoir to those in the cold reservoir. The heat flux is calculated according to the formula (3). Applying the formula (4), we can calculate the thermal conductivity along the zigzag direction of the h-BN membrane with a length of 21.1 nm is 392.71 (W/m–K). The thermal conductivity along the armchair direction of the h-BN membrane with a length of 20.9 nm is 370.73 (W/m–K).

The thermal conductivity of the workpieces with the length ranging from 20–90 nm is computed completely similar to the calculation of the thermal conductivity of the workpiece in Fig. 8a,c. The thermal conductivity of these workpieces at a temperature of 300 K is shown in Fig. 8e. We see that the size of the workpiece affects the thermal conductivity. As the length of the workpiece increases, the thermal conductivity increases. The influence of the workpiece length on the thermal conductivity of the system has been explained by previous studies due to the influence of the phonon scattering that existed at the boundary of the membrane. More phonons will be excited as the length increases, contributing to an increase in thermal conductivity^60^.

The thermal conductivity is proportional to the mean free path for phonon scattering according to the kinetic theory of phonon transport, and the thermal conductivity can be obtained by:

11 $$ \kappa = \frac{1}{3}CVl $$

where *C* and *V* denote the specific heat per volume and the average phonon velocity, *l* is the phonon mean free path. According to previous research^61^, in the situation of phonons scattering at the heat reservoir, the effective mean free path satisfies the relation:

12 $$ \frac{1}{{l_{eff} }} \propto \frac{1}{{l_{ph - ph} }} + \frac{1}{{l_{s} }} $$

where *l*_*ph-ph*_ and *l*_*s*_ denote the intrinsic phonon–phonon scattering length and the length of the simulation box.

According to Eqs. (11) and (12), the thermal conductivity satisfies the relation:

13 $$ \frac{1}{\kappa } \propto \frac{1}{{l_{ph - ph} }} + \frac{1}{{l_{s} }} $$

This indicates that a plot of the inverse of thermal conductivity, *κ*, against the inverse of system size, *l*_*s*_, should be a straight line. It also indicates that as the system length grows, the thermal conductivity grows.

Extrapolation of the NEMD results for h-BN samples with finite lengths, *κ*_*L*_, is a typical method for estimating the intrinsic thermal conductivity of h-BN monolayers with infinite lengths, *κ*_∞_. The length dependence of the thermal conductivity is proposed by Schelling et al.^62^,

14 $$ \frac{1}{\kappa (L)} = \frac{1}{{\kappa_{\infty } }}\left( {\frac{\lambda }{L} + 1} \right) $$

where *κ*_∞_is the intrinsic thermal conductivity for the structure with an infinite length (*L*∞), *L* is the sample length, and *λ* is the effective phonon mean free path.

The findings of thermal conductivity versus the inverse of sample length are shown in Fig. 8f. By linear fitting of *1/κ* and *1/L*, *κ*_∞_was determined by the extrapolation value when *1/L* → 0. The results show that the intrinsic thermal conductivity of the pristine h-BN sheet at 300 K in the zigzag and armchair directions is 518.13 W/m–K and 512.82 W/m–K, respectively.

**Figure 9:**
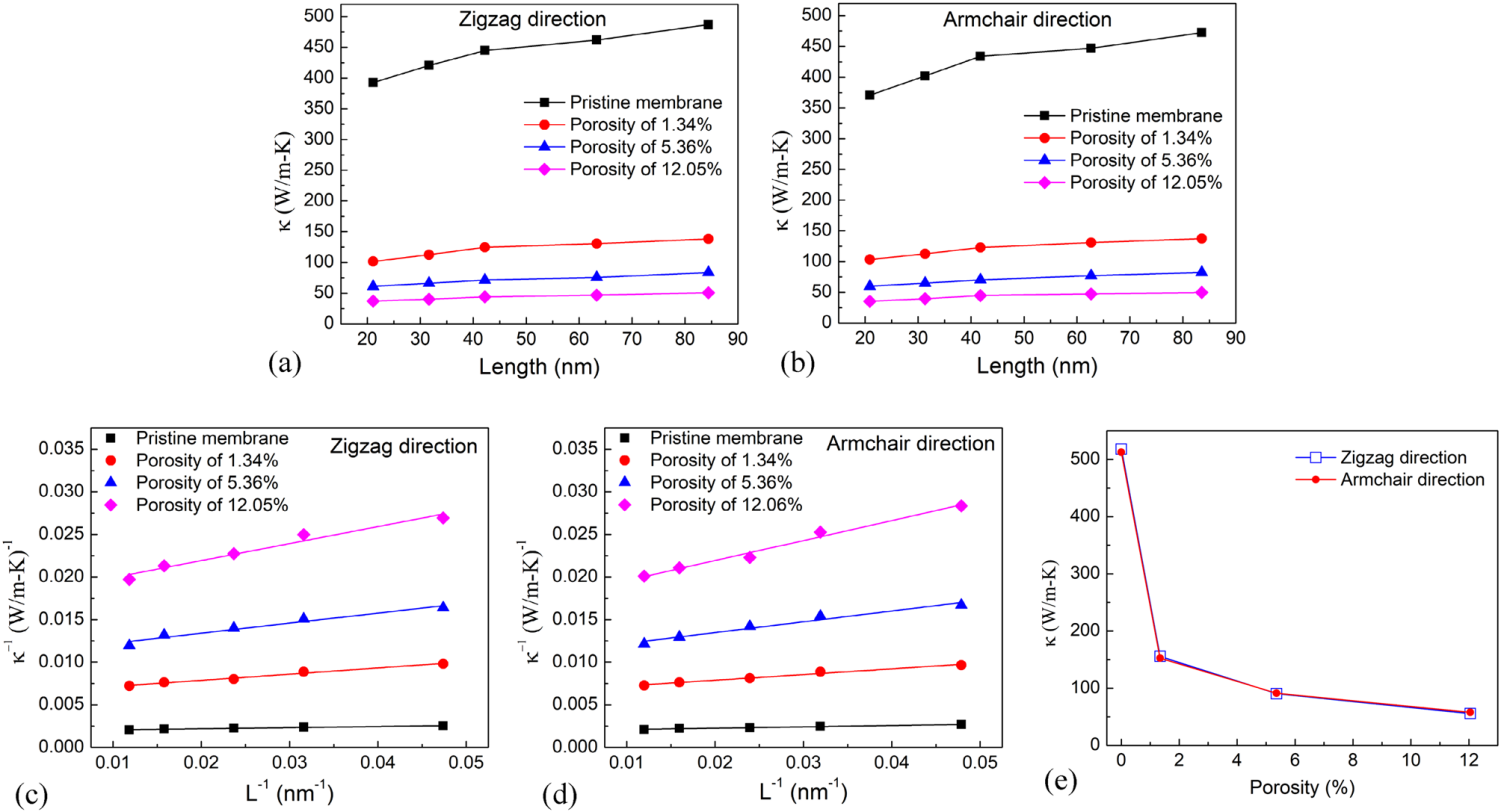
(**a,b**) Length dependence of thermal conductivity for single-layer h-BN membrane with various porosities in the zigzag and armchair directions. (**c,d**) Inverse of length versus inverse of thermal conductivity of single-layer h-BN membrane with various porosities in zigzag and armchair directions. **(e)** Porosity dependence of the intrinsic thermal conductivity of the single-layer h-BN membrane.

The length dependence of thermal conductivity for single-layer h-BN membrane with various porosities in the zigzag and armchair directions is shown in Fig. 9a,b. For each porous sheet, as the length of the sample increases, the thermal conductivity increases. When the length of the membrane is kept constant, the thermal conductivity of single-layer h-BN falls as the porosity increases from 0% to 12.5%. Figure 9c,d shows the system size dependence of *1/κ* on *1/L* of nanoporous h-BN membranes for the zigzag and armchair direction. The extrapolation value is used to derive the intrinsic thermal conductivities at various porosities when *1/L* → 0. Figure 9e shows the extrapolated thermal conductivity values of h-BN membranes with different porosities. It proves that the membrane's thermal conductivity is considerably affected by porosity. The thermal conductivity is reduced as porosity increases.

To further investigate the effect of porosity on the thermal conductivity of h-BN membranes, the vibration density of states (VDOS) of h-BN membranes is calculated. The VDOS is calculated by calculation of Fourier transform of atomic velocities autocorrelation function^41^:

15 $$ G\left( \omega \right) = \int {\left\langle {\sum\limits_{i = 1}^{N} {v_{i} (t)v_{i} (0)} } \right\rangle } e^{ - i\omega t} dt $$

where *N* denotes the number of atoms in the system, ω denotes the angular frequency, and *v*_*i*_*(t)* is the velocity of atom *i*-th at time *t*. $\left\langle {...} \right\rangle$ is atom number-averaged velocity autocorrelation function.

**Figure 10:**
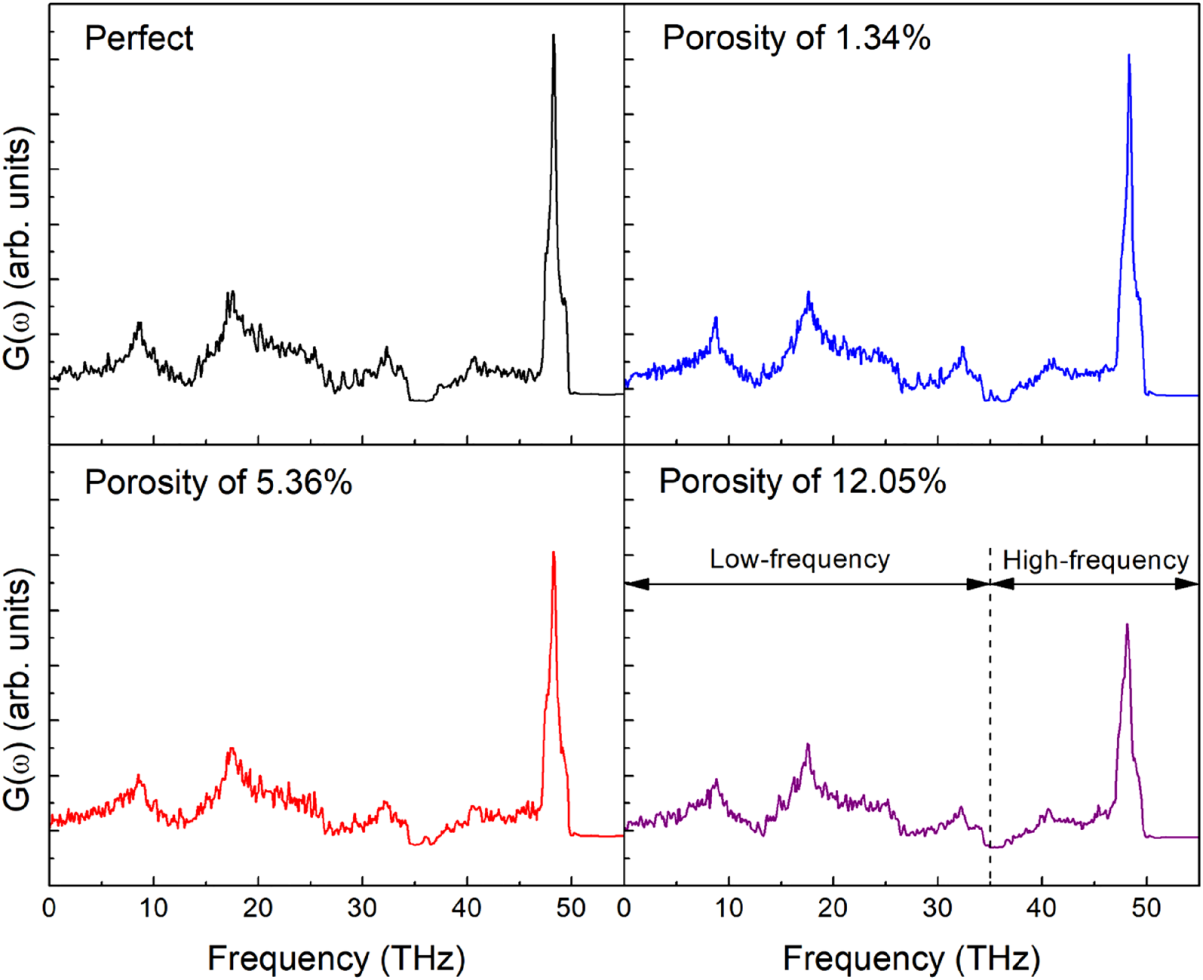
Phonon density of states of h-BN membranes.

To discuss the effect of porosity on the thermal conductivity, h-BN membranes of size 10.4 × 21.1 (W × L) nm^2^ with various porosities are used to calculate the vibration density of states at a temperature of 300 K. The calculated VDOS is illustrated in Fig. 10. As can be seen in the graph, porosity has a considerable impact on VDOS. The main peaks are about 48.3 THz in frequency. Thermal transport in the pristine h-BN is ballistic, which means phonon collisions are low. The main peaks' high of VDOS is decreased and slight shift towards low frequency as the porosity increase, implying that the defects might reduce the phonon mean free path due to high collision of the phonons. These alterations represent the scattering influence of defects on phonons, which reduces phonon life and considerably lowers thermal conductivity^63^. Phonon scattering has also been shown to be the predominant contributor in lowering the heat conductivity of other 2D materials^64^.

**Figure 11:**
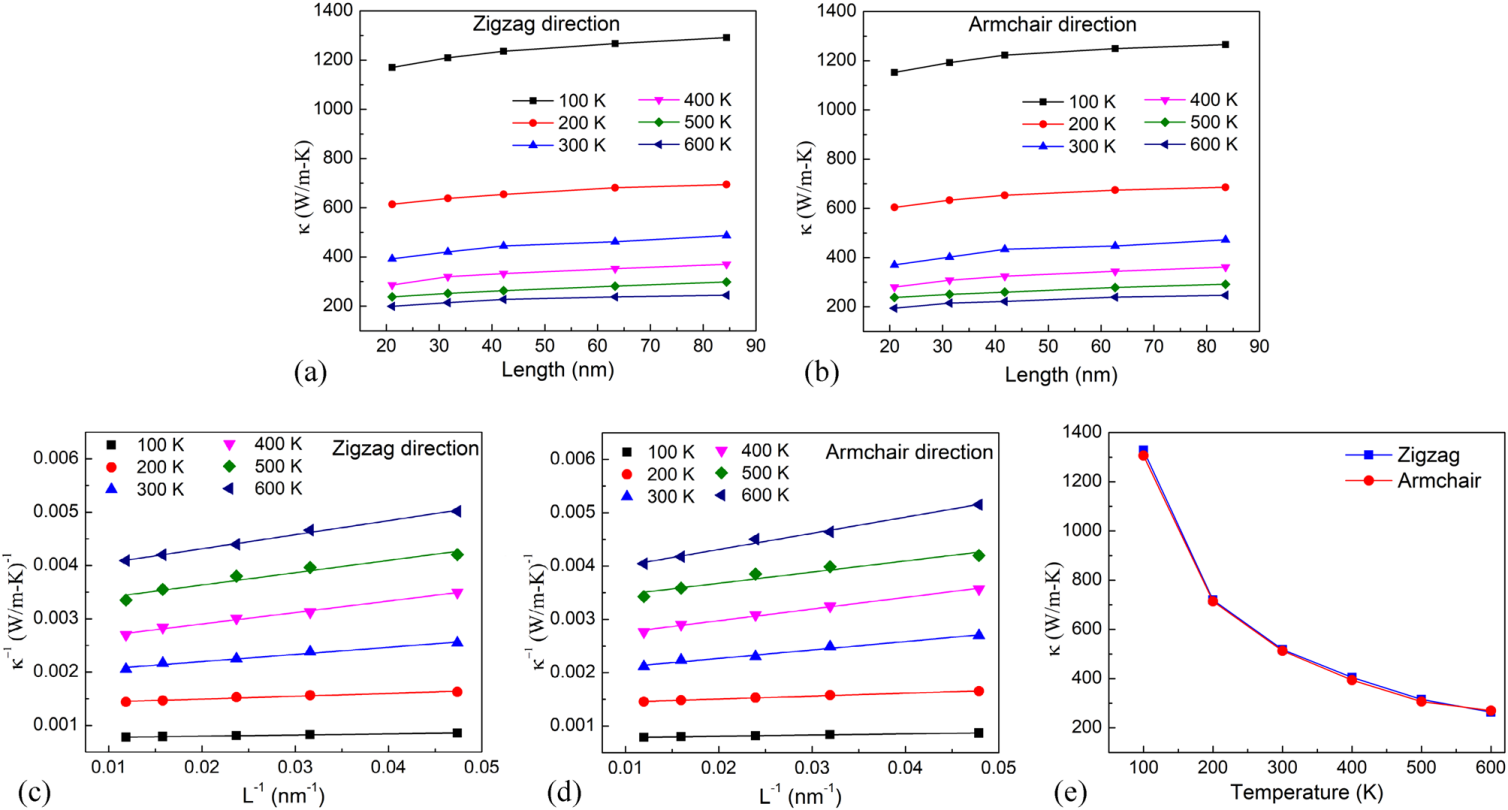
**(a,b)** System size dependence of thermal conductivity of single-layer h-BN at different temperatures. **(c,d)** System size dependence of *1/κ* on *1/L* of single-layer h-BN along the zigzag and armchair directions at different temperatures. **(e)** Temperature dependence of intrinsic thermal conductivities of h-BN membranes.

To address the temperature dependence of thermal conductivity of single-layer h-BN, we investigate with various temperatures varying from 100 to 600 K for pristine h-BN sheets with lengths varying from 21.1 up to 84.4 nm, as shown in Fig. 11a,b. Under the constant length condition of membranes, the thermal conductivity declines as the temperature rises. To estimate the intrinsic thermal conductivity of h-BN monolayer at various temperatures, the inverse of thermal conductivity versus the inverse of system length is plotted in Fig. 11c,d. From Fig. 11c,d, intrinsic thermal conductivity values of the h-BN membrane are extrapolated at various temperatures, as illustrated in Fig. 11e. The results present that the thermal conductivity of h-BN membranes in the armchair direction is very close in the zigzag direction. It clearly points out that the intrinsic thermal conductivity of h-BN reduces with the increase of temperature. This phenomenon has been explained in previous studies by Umklapp phonon–phonon scattering at high temperature^60^. The population of phonons with large momenta increases at high temperatures. When these large-moment phonons contact with other high-moment phonons, the total generated momenta readily exceeds the allowable momentum in the lattice structure, and they lose energy. As a result of the increased Umklapp scattering at high temperatures, the thermal transfer efficiency decreases.

To validate the computational model, we compare the results of the pristine membrane model with those of earlier investigations. Some mechanical and thermal characteristics values, including ultimate strength, fracture strain, and thermal conductivity, are guardedly addressed in Supplementary Table 1. The results show that the mechanical properties in this study are consistent with previous studies. Moreover, the thermal conductivity of h-BN thin films in this study is good consistent with experimentally investigated using the opto-thermal Raman technique^65^, and these values are in accordance with the theoretically predicted. As a consequence, the findings of our study on the influences of temperature and porosity on the mechanical characteristics and thermal conductivity of a single-layer h-BN membrane provide significant information that may be used in future h-BN research.

## Conclusions

In summary, this work investigates the effects of porosity and temperature on the mechanical characteristics of h-BN membranes under uniaxial and biaxial tensions. It is found that the porosity and temperature significantly affect the tensile characteristic of h-BN membranes. Depending on the porosity and tensile direction, the phase transition occurs more or less. As porosity increases, the strength and Young's modulus decrease. The findings reveal that the strength, Young's modulus, also reduce as temperature increases. It is interesting to see that the cracks preferentially spread in the zigzag edge under deformation in the zigzag or armchair direction. In addition, this work conduct NEMD simulations to study the influence of temperature and porosity on the thermal conductivity of h-BN membranes. The results reveal that the thermal conductivity is greatly reduced by nanoporous. The higher the porosity, the lower the thermal conductivity. The vibration density of states of h-BN membranes is calculated; the result suggests that the defects might reduce the phonon mean free path because of the high collision of the phonons. These alterations represent the scattering influence of defects on phonons, which reduces phonon life and considerably lowers thermal conductivity. Moreover, the findings also proved that as temperature increases, the intrinsic thermal conductivity of h-BN decreases.

## Supplementary Information

Supplementary Information 1.

Supplementary Information 2.

## Data Availability

All files for LAMMPS can be found in the Supplemental materials. The datasets used and/or analysed during the current study available from the corresponding author on reasonable request.
