# Supplementary material for: Mechanical and thermal characterizations of nanoporous two-dimensional boron nitride membranes
Source: Sci Rep. 2022 Apr 15;12:6306. doi: 10.1038/s41598-022-10424-4 (PMC9012750; doi:10.1038/s41598-022-10424-4)
Supplement: Supplementary file 2 — Supplementary Information 2. [file 41598_2022_10424_MOESM2_ESM.docx]

**Supplementary Materials**

**“Mechanical and thermal characterizations of nanoporous two-dimensional boron nitride membranes”**

**Fig. S1.** Stress-strain curves of the h-BN membrane with various porosity at a temperature of 300 K in biaxial tension. (a) Stress-strain curves in the zigzag direction as the h-BN membranes are under biaxial tensile loading. (b) Stress-strain curves in the armchair direction as the h-BN membranes are under biaxial tensile loading.

**Fig. S2.** The von Mises stress distribution and the fracture evolution of h-BN membranes under biaxial tension at 300 K with different porosities: (a) 1.34%, (b) 5.36%, (c) 12.05%.

**Fig. S3.** Stress-strain curves of the pristine h-BN membrane at various temperatures in biaxial tension. (a) Stress-strain curves in the zigzag direction as the h-BN membranes are under biaxial tensile loading. (b) Stress-strain curves in the armchair direction as the h-BN membranes are under biaxial tensile loading.

**Fig. S4.** Stress-strain curves of the pristine h-BN membrane at various strain rates in uniaxial tension. (a) Stress-strain curves in the zigzag direction. (b) Stress-strain curves in the armchair direction.

**Supplementary Table 1**

Comparison of the calculated results and relevant values obtained by previous works

| Material | Young’s modulus  (GPa) | Strength (GPa) | Thermal conductivity (W/m-K) | Temperature | Layer thickness  (nm) | References |
| --- | --- | --- | --- | --- | --- | --- |
| h-BN | 665 | - | - | 0 K | 0.42 | Oliveira et al. [66] |
| h-BN | - | 86.16 | - | 0 K |  | Peng et al. [67] |
| h-BN | 678 (zigzag)  611 (armchair) | 133  116 |  | 300 K | 0.33 | Ding  et al. [68] |
| h-BN | 692.7 (zigzag)  739.9 (armchair) | 114.1  126.6 |  | 300 K | 0.33 | Zhao  et al. [49] |
| h-BN |  |  | 450 | 300 K | 0.335 | Kinaci et al. [45] |
| h-BN |  |  | 600 | 300 K | 0.33 | Lindsay et al. [69] |
| h-BN |  |  | 545 | 315 K | 0.33 | Ying et al. [65] |
| h-BN | 630 | 104 | 550±60 | 300 K | 0.333 | Mahdizadeh et al. [70] |
| h-BN |  |  | 606 | 300 K | 0.33 | Wang et al. [71] |
| h-BN | 712.8 (zigzag)  708.1 (armchair) | 113.42  108.91 | 518.13  512.82 | 300 K  300 K | 0.33 | This research |

**Supplementary references**

[66] Oliveira, I. S., Lima, J. S., Freitas, A., Bezerra, C. G., Azevedo, S., & Machado, L. D. (2021). Investigating size effects in graphene–BN hybrid monolayers: a combined density functional theory-molecular dynamics study. *RSC Advances*, *11*(21), 12595-12606.

[67] Peng, Q. (2018). Strain-induced dimensional phase change of graphene-like boron nitride monolayers. *Nanotechnology*, *29*(40), 405201.

[68] Ding, Q., Ding, N., Liu, L., Li, N., & Wu, C. M. L. (2018). Investigation on mechanical performances of grain boundaries in hexagonal boron nitride sheets. *International Journal of Mechanical Sciences*, *149*, 262-272.

[69] Lindsay, L., & Broido, D. A. (2011). Enhanced thermal conductivity and isotope effect in single-layer hexagonal boron nitride. *Physical Review B*, *84*(15), 155421.

[70] Mahdizadeh, S. J., Goharshadi, E. K., & Akhlamadi, G. (2016). Thermo-mechanical properties of boron nitride nanoribbons: A molecular dynamics simulation study. *Journal of Molecular Graphics and Modelling*, *68*, 1-13.

[71] Wang, Y., Chang, Z., Gao, K., Li, Z., Hou, G., Liu, J., & Zhang, L. (2021). Designing high thermal conductivity of polydimethylsiloxane filled with hybrid h-BN/MoS2 via molecular dynamics simulation. *Polymer*, *224*, 123697.
